# A novel DNA vaccine expressing the Ag85A-HA2 fusion protein provides protection against influenza A virus and *Staphylococcus aureus*

**DOI:** 10.1186/1743-422X-10-40

**Published:** 2013-01-31

**Authors:** Jun Dai, Decui Pei, Baoning Wang, Yu Kuang, Laifeng Ren, Kang Cao, Bin Zuo, Jingjing Shao, Sha Li, Zhonghua Jiang, Hong Li, Mingyuan Li

**Affiliations:** 1 Department of Microbiology, West China School of Preclinical and Forensic Medicine, Sichuan University, Chengdu, Sichuan 610041, PR China; 2 Key Laboratory of Obstetric & Pediatic Disease and Birth Defects of Ministry of Education, West China Second University Hospital, Sichuan University, Chengdu, Sichuan, 610041, China; 3 State Key Laboratory of Oral Diseases, Sichuan University, Chengdu, Sichuan 610041, PR China

**Keywords:** *Staphylococcus aureus*, Influenza a viruse, *Mycobacterium tuberculosis* secreted antigen Ag85A, DNA vaccine

## Abstract

Secondary pneumonia due to *Staphylococcus aureus* (*S*. *aureus*) causes significant morbidity and mortality. The aim of the research was designed a novel DNA vaccine encoding the *Mycobacterium tuberculosis* secreted antigen Ag85A fused with the influenza A virus (IAV) HA2 protein to provide protection against both influenza and secondary infection with *S*. *aureus*. The DNA vaccine vector efficiently expressed the encoded antigen in mammalian cells, as determined by RT-PCR, Western blotting and immunofluorescence analysis. Mice were immunized with the vaccine by intramuscular injection before challenge with IAV and *S*. *aureus*. The pulmonary and the splenocyte culture IFN-γ levels were significant higher in immunized mice than their respective controls. Although the antibody titer in the HI test was low, the sera of mice immunized with the novel vaccine vector were effective in neutralisation assay *in vitro*. The vaccine could reduce the loss of body weight in mice during IAV challenge. Both Western blotting and RT-PCR showed that the vaccine markedly enhanced toll like receptor 2 (TLR2) expression in splenocytes after the secondary infection with *S*. *aureus*. The survival rate of mice with high TLR2 expression (pEGFP/Ag85A-HA2 or iPR) was significantly increased compared with mice immunized with pEGFP/HA2 after challenge with *S*. *aureus*. However, the pulmonary IL-10 concentration and *S*. *aureus* titer were significantly decreased in immunized mice, and expression of TLR2 was increased after challenge with *S*. *aureus*. These results demonstrated that Ag85A could strengthen the immune response to IAV and *S*. *aureus*, and TLR2 was involved in the host response to *S*. *aureus*.

## Introduction

Secondary pneumonia due to *Staphylococcus aureus* (*S*. *aureus*) is a significant cause of mortality associated with influenza A virus (IAV) infection [1,2]. Antibody responses against the viral surface protein hemagglutinin (HA) are major determinants for protection against IAV [3]. However, variations in HA occur rapidly due to antigenic shift and drift, allowing the virus to evade immunity conferred by seasonal vaccines [4]. Therefore, development of an IAV vaccine to enhance immune responses to conserved domains of IAV and *S*. *aureus* would be highly advantageous. HA is synthesized as a precursor HA0, which is cleaved into the HA1 and HA2 subunits. While the membrane distal domain (HA1) can be highly variable, the stalk region of HA, which contains the core fusion machinery constituted primarily by the HA2 subunit, is relatively conserved. Antibodies targeting the HA2 subunit can provide broad protection against both seasonal and pandemic influenza A infections [5]. However, weak antigenicity [6] is one of the reasons that outbreaks of IAV and secondary pneumonia cannot be prevented by vaccination with the fusion peptide alone. It would therefore be beneficial for a vaccine to enhance immune responses against both IAV and *S*. *aureus* infections.

The Bacille Calmette-Guérin (BCG) vaccine against *Mycobacterium tuberculosis* has been shown to have a marked immunomodulatory effect in combination with influenza vaccines and can enhance antigen-specific antibody production. Sera from mice vaccinated with BCG exhibit antibacterial activity [7]. Ag85A, as a major secreted antigen of *M*. *tuberculosis* and an immunodominant antigen of BCG, can increase T helper type 1 (Th1) cytokine responses [8]. The Th1 cytokine, interferon (IFN)-γ, can upregulate expression of toll-like receptor 2 (TLR2), which recognizes Staphylococcal peptidoglycans and induces activation of immune responses [9]. As it is possible that Ag85A can upregulate TLR2 expression and immune activation has been shown to offer protection against *S*. *aureus*, we therefore hypothesized that immune reactions to Ag85A may be able to eliminate this bacteria [10,11]. Although the upregulation of TLR2 may be helpful against bacterial infection, it has been suggested that TLR2 does not contribute to host responses during post-influenza pneumonia [12], Therefore, it is not yet clear whether upregulation of TLR2 would be beneficial in such a scenario.

We constructed a novel influenza vaccine construct expressing a conserved region of the HA protein linked with Ag85A to evaluate whether *M*. *tuberculosis* secreted antigen Ag85A may serve as a good immune adjuvant for HA2. The efficacy of this vaccine in preventing morbidity after challenge with IAV and mortality in mice was evaluated after challenge with IAV and *S*. *aureus*, and cytokine profiles were determined in lung homogenates and cultured splenocytes. We also explored the role of TLR2 upregulation in post-influenza pneumonia.

## Results

### Expression of Ag85A and/or HA2 from plasmid DNA vectors in eukaryotic cells

**Figure 1:**
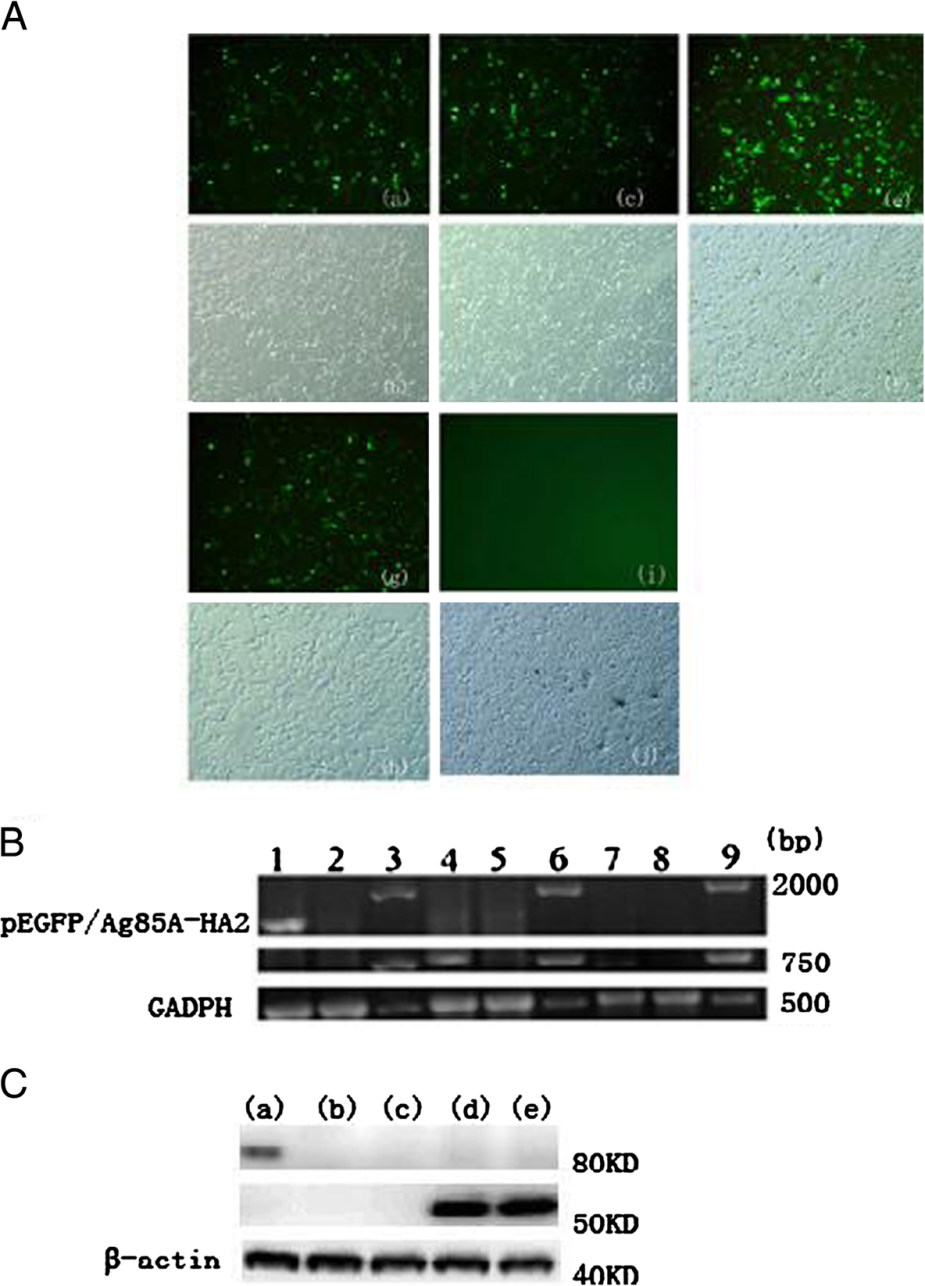
**Expression of HA2 or Ag85A in HEK293 cells. (A) Expression of HA2 or Ag85A in HEK293 cells.** HEK293 cells were transfected with plasmids encoding individual HA2 or Ag85A proteins or the Ag85A-HA2 fusion protein, and expression was evaluated by GFP fluorescence. Shown are cells transfected with pEGFP/HA2 (a, b), pEGFP/Ag85A (c, d), pEGFP/C2 (e, f), pEGFP/Ag85A-HA2 (g, h) or untransfected cells (i, j). Fluorescent images (a, c, e, g, i) and phase contrast images (b, d, f, h, j) are shown. **(B)** Expression was evaluated by RT-PCR. (7) cells transfected with pEGFP/ HA2; (3,6,9) Trans2K Plus DNA Marker; (2,5,8) cells transfected with pEGFP-C2; (4) cells transfected with pEGFP/Ag85A; (1) cells transfected with pEGFP/ Ag85A-HA2. Fragments of approximately 1500 bp (1), 750 bp (4, 7) and 500 bp (1, 2, 4, 5, 7 and 8) were separated by 2% agarose gel electrophoresis following RT-PCR. GADPH: Fragments of approximately 500 bp; pEGFP/Ag85A: Fragments of approximately 750 bp; pEGFP/ HA2: Fragments of approximately 750 bp; pEGFP/ Ag85A-HA2: Fragments of approximately 1500 bp. **(C)** Expression was evaluated by Western blotting. (a) cells transfected with pEGFP/ Ag85A-HA2; (b) cells transfected with pEGFP-C2; (c) cells transfected with PBS; (d) cells transfected with pEGFP/ HA2; (e) cells transfected with pEGFP/Ag85A.

pEGFP/Ag85A-HA2, pEGFP-C2, pEGFP/Ag85A and pEGFP/HA2 were successfully transfected in HEK293 cells. Strong green fluorescent signals were clearly seen in the cells transfected with all four plasmids (Figure 1A). The results of RT-PCR (Figure 1B) and western blotting (Figure 1C) also confirmed that pEGFP/Ag85A-HA2, pEGFP/Ag85A and pEGFP/HA2 successfully expressed the expected transcripts in transfected HEK293 cells. Therefore, the Ag85A, HA2 and the Ag85A-HA2 fusion genes could be transiently expressed in HEK 293 cells.

### Comparison of antibody titers and influenza virus loads

The mice were inoculated with pEGFP/Ag85A-HA2, pEGFP/HA2, pEGFP/Ag85A, PBS, pEGFP/C2 or iPR. Development of antibodies against HA was evaluated by hemagglutination inhibition (HI) assays. The HI titers were represented as group means of log 2 dilution. As shown in Table 1, the highest HI titer against PR8 virus was detected in mice immunized with iPR, which increased by approximately 3-fold after boosting. However, HI activity against PR8 virus was not detected in mice immunized with pEGFP/HA2 or pEGFP/Ag85A-HA2. The HI antibody titers were low (<10) in these groups both in the primary response and in the booster response, as might be expected based on the absence of the HA1 antigen in the vaccine which could induce antibodies to the receptor binding site or other epitopes involved in HI.

**Table 1:** Comparison of influenza virus load and antibody titers

**Group**	**Lung virus titer (log10 TCID50)**	**Antibody titer (HI assay)**	
		**Primary response**	**Secondary response**
inactivated PR	2.17 ± 0.58	9 ± 5.03	24 ± 9.24
pEGFP/Ag85A-HA2	3.67 ± 0.77	4.5 ± 2.52	8 ± 5.66
pEGFP/Ag85A	6.17 ± 0.57	1.5 ± 1.91	2.5 ± 1.71
pEGFP/HA2	4.67 ± 0.98	3 ± 1.15	4.5 ± 2.52
pEGFP/C2	6.83 ± 0.94	0.5 ± 1.00	0.5 ± 1.00
PBS	6.92 ± 0.79	0.5 ± 1.00	0.5 ± 1.00

**Figure 2:**
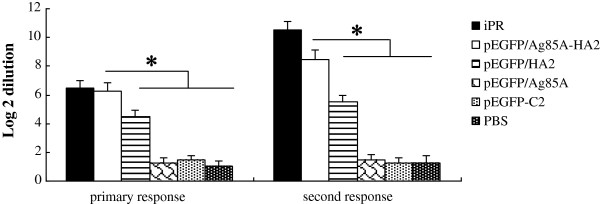
Comparison of serum antibody titers (log 2 dilution) giving 50% CPE inhibition assay in MDCK cells. (*P **< 0.05).** The high NAb titers were detected in mice immunized with iPR, pEGFP/Ag85A-HA2 and pEGFP/HA2. The NAb titer from the pEGFP/Ag85A-HA2 vaccinated group was higher than those of the pEGFP-C2, pEGFP/HA2 and pEGFP/Ag85A vaccinated groups (P < 0.05). The NAb titer of the pEGFP/HA2 vaccinated group was higher than those of the pEGFP-C2, PBS and pEGFP/Ag85A vaccinated groups (P < 0.05).

Development of neutralizing antibodies (NAbs) against HA was evaluated by the neutralisation assay. The titer of each serum sample was expressed as the dilution (log 2 transformed) that decreased the CPE due to the inoculating virus by 50% in MDCK cells. As shown in Figure 2, The NAb titer of the pEGFP/HA2 vaccinated group was higher than those of the pEGFP-C2, PBS and pEGFP/Ag85A vaccinated groups (*P* < 0.05). High NAb titers were detected in mice immunized with iPR, pEGFP/Ag85A-HA2 and pEGFP/HA2. The NAb titer from the pEGFP/Ag85A-HA2 vaccinated group was higher than those of the pEGFP-C2, pEGFP/HA2 and pEGFP/Ag85A vaccinated groups (*P* < 0.05).

However, influenza virus loads decreased in mice immunized with iPR, pEGFP/HA2 or pEGFP/Ag85A-HA2 (Table 1) (*P* < 0.05). The influenza virus titers of samples from the pEGFP/Ag85A-HA2 vaccinated group was lower than that of the pEGFP-C2, pEGFP/HA2 and pEGFP/Ag85A vaccinated groups (*P* < 0.05).

### Cytokine measurements

**Figure 3:**
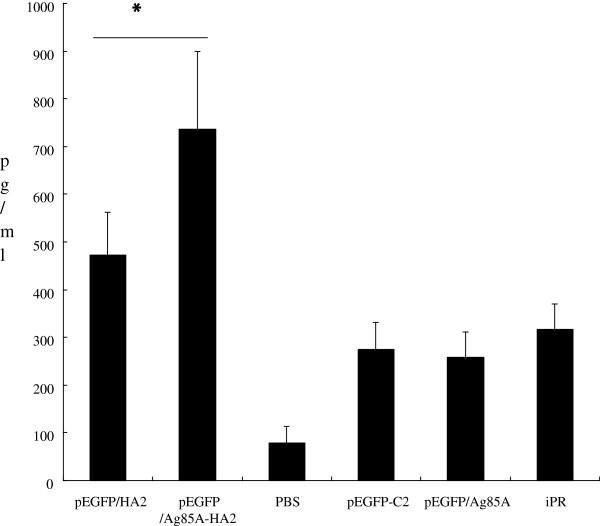
IFN-γproduction in splenocyte culture 72 h after induced by HA in vitro. *P **< 0.05.** HA induced higher IFN-γ titers in the pEGFP/Ag85A-HA2 group (*P* < 0.05). Splenocytes from mice vaccinated with pEGFP/Ag85A-HA2 produced 1.5-fold higher IFN-γ in response to HA than those from mice vaccinated with pEGFP/HA2 (*P* < 0.05).

*In vitro* IFN-γ production was evaluated 8 days after the virus challenge. IFN-γ was induced by HA or conA *in vitro*. IFN-γ production in the PBS group was markedly lower than that of other groups when induced by HA (*P* < 0.05). However, HA induced higher IFN-γ titers in the pEGFP/Ag85A-HA2 group (Figure 3) (*P* < 0.05). Splenocytes from mice vaccinated with pEGFP/Ag85A-HA2 produced 1.5-fold higher IFN-γ in response to HA than those from mice vaccinated with pEGFP/HA2 (*P* < 0.05). HA induced markedly lower IFN-γ levels than did conA (data not shown).

**Figure 4:**
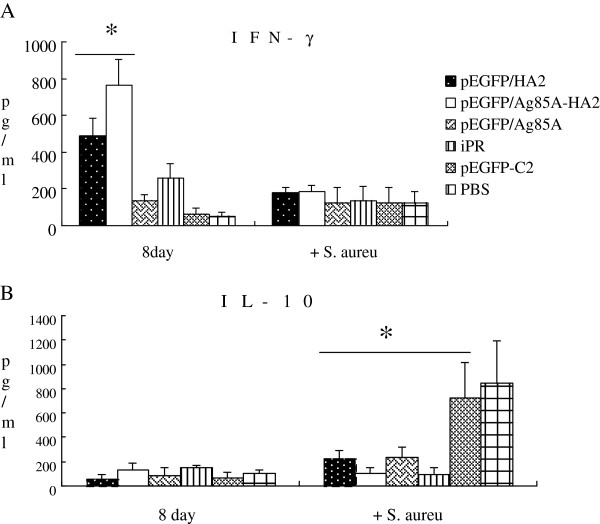
**Cytokine levels in mouse lung homogenates.** IFN-γ (*P < 0.05) (**A**) and IL-10 (*P < 0.05) (**B**) of lung homogenates day 8 after infection with A/PR/8/34 and on day 2 after infection with *S*. *aureus*. The pulmonary IFN-γ levels of mice vaccinated with pEGFP/Ag85A-HA2 were significantly higher than those vaccinated with pEGFP/HA2 on day 8 after infection with A/PR/8/34 (P < 0.05). The pulmonary IFN-γ levels of mice vaccinated with pEGFP/HA2 were significantly higher than those vaccinated with pEGFP-C2 or PBS (*P* < 0.05). The pulmonary levels of the anti-inflammatory cytokine IL-10 were significantly higher in the PBS group and the pEGFP-C2 vaccinated group than those in the other groups at day 2 after infection with *S*. *aureus* (*P* < 0.05).

We measured IL-10 and IFN-γ in lung homogenates on day 8 after infection with A/PR/8/34 and at day 2 after infection with *S*. *aureus*. Some differences were found between the pEGFP/Ag85A-HA2 group and other groups. In particular, the pulmonary IFN-γ levels were significantly higher in the pEGFP/Ag85A-HA2 vaccinated group on day 8 after infection with A/PR/8/34 (Figure 4A). The pulmonary IFN-γ levels of mice vaccinated with pEGFP/Ag85A-HA2 were significantly higher than those vaccinated with pEGFP/HA2 on day 8 after infection with A/PR/8/34 (*P* < 0.05). The pulmonary IFN-γ levels of mice vaccinated with pEGFP/HA2 were significantly higher than those vaccinated with pEGFP-C2 or PBS (*P* < 0.05). The pulmonary levels of the anti-inflammatory cytokine IL-10 were significantly higher in the PBS group and the pEGFP-C2 vaccinated group than those in the other groups at day 2 after infection with *S*. *aureus* (*P* < 0.05). However, the levels of IL-10 were lower in the pEGFP/Ag85A-HA2 vaccinated group on day 2 after infection with S. aureus (P < 0.05) (Figure 4B).

### Expression of TLR2

**Figure 5:**
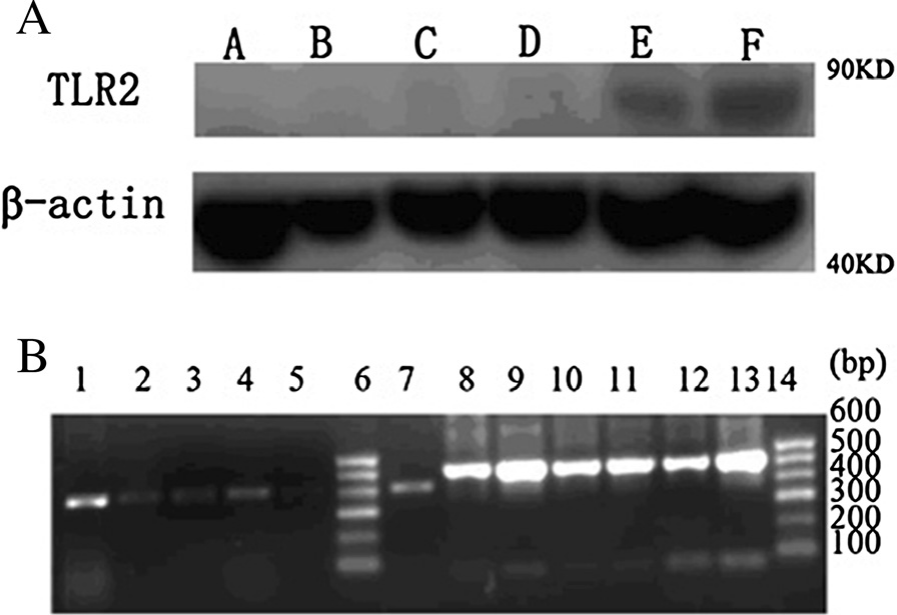
**Expression of TLR2 in splenocytes of different vaccinated groups on day 2 after infection with *S*. *aureus*. (A)** Western blot detection of TLR2 in splenocytes. Lane A: PBS group; lane B pEGFP-C2 group; lane C pEGFP /HA2 group; lane D pEGFP/Ag85A group; lane E pEGFP/Ag85A-HA2 group; lane F iPR group. **(B)** RT-PCR detection of TLR2 in splenocytes. Lanes 1, 8: pEGFP/Ag85A-HA2 group; lanes 2, 9: pEGFP-C2 group; lanes 3, 10: pEGFP /HA2 group; lanes 4, 11: pEGFP/Ag85A group; lanes 5, 12: PBS group; lanes 7, 13: iPR group. Lanes 1–5, 7: TLR2 (410 bp); lanes 8–13: GADPH (531 bp); lanes 6, 14: marker.

In vivo expression of TLR2 was evaluated by Western blotting and RT-PCR 2 days after *S*. *aureus* challenge. Splenocyte TLR2 expression in mice vaccinated with pEGFP/Ag85A-HA2 or iPR was higher than those in their respective controls, and there was no difference between the pEGFP/Ag85A-HA2 group and iPR group (Figure 5A,B).

### Comparison of body weight loss, survival rate and bacterial outgrowth during post-influenza pneumonia

**Figure 6:**
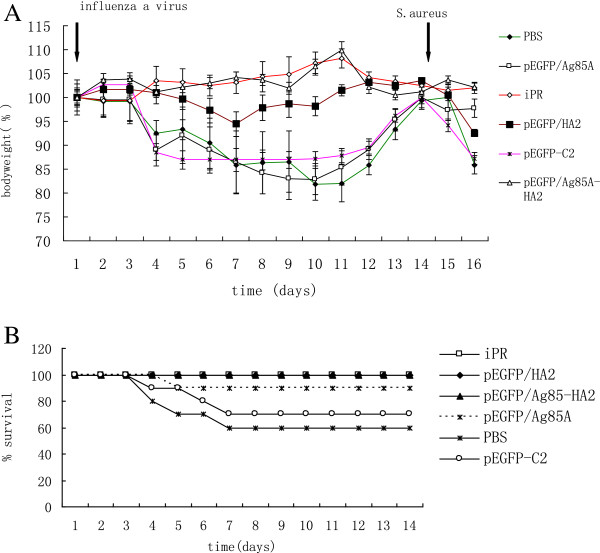
**A Body weight of mice.** Weight was calculated relative to day 0. Data are mean ± SEM of four mice per group. **B** Survival rate of mice after infection with IAV. Survival rate was calculated from infection with IAV to infection with *S*. *aureus*.

As IAV infection can induce body weight loss, it can be used to follow the course of infection in mice. At 14 days after infection with influenza, mice of all groups were intranasally inoculated with *S*. *aureus*. Marked body weight loss was detected 48 h after infection. Body weight decreased obviously in the PBS, pEGFP/HA2, pEGFP/Ag85A and pEGFP-C2 groups. The body weight of the pEGFP/Ag85A-HA2 and iPR groups did not change (*P* < 0.05 vs. mice of PBS group, pEGFP/HA2 group, pEGFP/Ag85A group and pEGFP/C2 group) (Figure 6A). And survival rate decreased obviously in the PBS and pEGFP-C2 groups after infection with IAV (Figure 6B).

**Figure 7:**
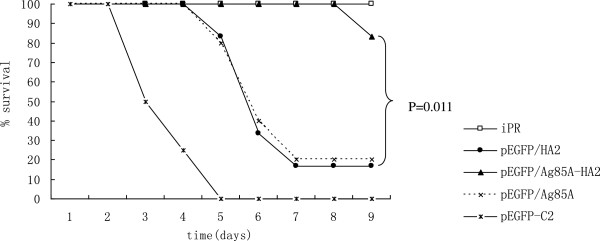
**Survival rate of mice from day 3 after infection with *S*. *aureus*.** The survival rate of mice vaccinated with pEGFP/Ag85A-HA2 or iPR was significantly higher than their respective controls (*P* < 0.05).

The survival rate was monitored from day 3 after infection with *S*. *aureus*. In the PBS group, only two mice survived for 3 days and no mice survived 4 days after *S*. *aureus* challenge (data not shown). The survival rate was high in the pEGFP/Ag85A-HA2 and iPR groups, while it was low in other groups. There were significant differences in the survival rates between the pEGFP/Ag85A-HA2 group or iPR group and other groups (*P* < 0.05) (Figure 7). The survival rate of mice vaccinated with pEGFP/Ag85A-HA2 was significantly higher than that of mice vaccinated with pEGFP/HA2 (*P* < 0.05). The survival rate of mice vaccinated with pEGFP/HA2 was significantly higher than that of mice vaccinated with pEGFP-C2 or PBS. Additionally, we did not find a significant difference between the pEGFP/Ag85A-HA2 group and iPR group (*P* > 0.05) (Figure 7).

**Figure 8:**
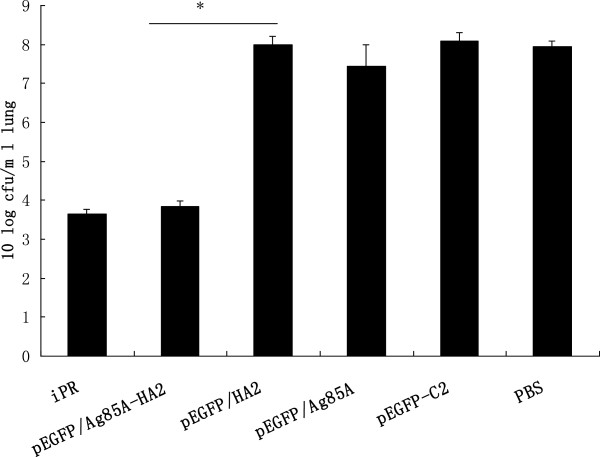
Bacterial outgrowth during postinfluenza pneumonia. (*P **< 0.05). ***S*. *aureus* titration was performed on day 2 after infection with *S*. *aureus*. The *S*. *aureus* titer in mice vaccinated with pEGFP/Ag85A-HA2 was lower than that in mice vaccinated with pEGFP/HA2.

*S*. *aureus* titration was performed to further assess the host defense against secondary Staphylococcal infection. The *S*. *aureus* titer in mice vaccinated with pEGFP/Ag85A-HA2 or iPR was lower than that in mice vaccinated with pEGFP/HA2, pEGFP/Ag85A or PBS (*P* < 0.05) (Figure 8).

## Discussion

Variability of IAV is one of the difficulties in developing prophylactic vaccines. Once infected with IAV, secondary respiratory tract infections caused by *S*. *aureus* is also problematic [13-15]. Post-influenza staphylococcal pneumonia is associated with a strong inflammatory response in the lungs [16,17]. Therefore, it is important for novel influenza virus vaccines to provide protection from *S*. *aureus* as well.

In this study, we developed a DNA vaccine encoding the Ag85A-HA2 fusion protein with the aim of inducing protection against both IAV and *S*. *aureus*. After confirming that they could express successfully in mammalian cells, the novel vaccine and respective controls were used to immunize mice by the intramuscular route before challenge with IAV and *S*. *aureus*. It was interesting that while the antibody titer in the HI test was low, the sera of mice immunized with the novel vaccine vector were effective in neutralisation assay in vitro. The vaccine could reduce the loss of body weight in mice during IAV challenge. This observation is supported by a previous study describing neutralizing antibodies to HA2 which could not completely prevent hemagglutination of erythrocytes [5]. These results demonstrated that the immune responses to HA2 was successfully induced by the pEGFP/Ag85A-HA2 vaccine. Further tests are warranted to determine whether these responses to HA2 may confer protection against diverse IAV.

IFN-γ is a representative Th1 cytokine associated with inhibition of IAV proliferation. Analysis of cytokine production showed that the pulmonary IFN-γ levels in the pEGFP/HA2 group were significantly higher than those of the pEGFP-C2 and PBS groups after IAV challenge. HA induced higher IFN-γ titers in the pEGFP/Ag85A-HA2 group and pEGFP/ HA2 group (*P* < 0.05). Splenocytes from mice vaccinated with pEGFP/Ag85A-HA2 produced 1.5-fold higher IFN-γ in response to HA than those from mice vaccinated with pEGFP/HA2 (*P* < 0.05). The weight loss of mice vaccinated with pEGFP/HA2 was lower than that of mice vaccinated with pEGFP-C2 or PBS after IAV challenge. The survival rate of mice vaccinated with pEGFP/HA2 was higher than that of mice vaccinated with pEGFP-C2 or PBS. However, the weight loss after IAV challenge and the survival rate after *S*. *aureus* challenge in the pEGFP/HA2 group were lower than those in the iPR group. These result confirmed that HA2 could provide limited protection against IAV and *S*. *aureus* challenge.

The cytokine analysis also showed that the pulmonary IFN-γ levels in the pEGFP/Ag85A-HA2 vaccinated group were significantly higher than that in the pEGFP/HA2 vaccinated group. Splenocytes from mice vaccinated with pEGFP/Ag85A-HA2 produced 1.5-fold higher IFN-γ in response to HA than those from mice vaccinated with pEGFP/HA2 (*P* < 0.05). These results showed that pEGFP/Ag85A-HA2 could induce high Th1 response. The weight loss of mice vaccinated with pEGFP/Ag85A-HA2 was lower than that of mice vaccinated with pEGFP/HA2. The IAV titer in the pEGFP/Ag85A-HA2 group was lower than those of the pEGFP-C2, pEGFP/HA2 and pEGFP/Ag85A group. These results demonstrated that Ag85A could enhance the protective effects against IAV. The survival rate after *S*. *aureus* challenge in the pEGFP/Ag85A-HA2 group was higher than that in the pEGFP/HA2 group. The *S*. *aureus* titers of the pEGFP/Ag85A-HA2 group and iPR group were lower than that of the pEGFP/HA2 group. The higher survival rate and the lower *S*. *aureus* titer after challenge in the pEGFP/Ag85A-HA2 group was probably due to Ag85A-mediated immune activation, which enhanced the protective effects of HA2 against IAV challenge. The increased protection against IAV challenge decreased the influenza virus titer. As IAV can suppress the functions of immune cells [18,19], it was possible that the elimination of IAV could enhance those functions. In other words, the decreased influenza virus titer may have enhanced immune protection against *S*. *aureus* challenge.

TLR2 is considered important for sensing gram-positive bacteria [20,21], and its upregulation has been positively associated with increased concentrations of Gram-positive bacteria [22]. TLR2 enhances neutrophil activity of killing of *S*. *aureus* and promotes clearance of *S*. *aureus*[10]. However, since TLR2 may not contribute to host responses during post-influenza pneumonia [12], it was previously not clear whether increasing TLR2 expression would prevent or ameliorate this condition. Our results here showed that TLR2 expression in mice vaccinated with pEGFP/Ag85A-HA2 or iPR was also higher than that of mice vaccinated with pEGFP/HA2. The survival rate of mice with high TLR2 expression was significantly increased after challenge with *S*. *aureus*.

IL-10 is an important mediator of the enhanced susceptibility to staphylococcal pneumonia [23]. A high level of IL-10 can result in increased bacterial outgrowth and markedly increased lethality during secondary bacterial pneumonia. In our study the IL-10 levels in lung homogenates were significantly lower in the pEGFP/Ag85A-HA2 vaccinated group and the iPR vaccinated group than those of other groups at day 2 after infection with *S*. *aureus* (*P* < 0.05). These results demonstrated that TLR2 was involved in the host response to *S*. *aureu*s and enhanced elimination of the bacteria, thereby reducing the incidence of post-influenza pneumonia.

Interactions between TLR2 and their ligands typically results in cellular activation and the production of inflammatory cytokines. IL-10 could inhibit production of inflammatory cytokines by inhibition of MyD88 pathway. It is possible that IL-10 inhibit immune reaction by affecting the expression of TLR2. Infection of IAV could increase the level of IL-10. Our results here showed that TLR2 expression in mice vaccinated with pEGFP/Ag85A-HA2 or iPR was also higher than pEGFP-C2 and IL-10 level in mice vaccinated with pEGFP/Ag85A-HA2 or iPR was lower than that of mice vaccinated with pEGFP-C2. These results demonstrated that IL-10 could affect the expression of TLR2.

BCG vaccine has been used widely. Ag85A is an immunodominant antigen of BCG. Therefore, the immune reactions against Ag85A could be easily induced. Our results showed that Ag85A could enhance immune responses to HA2. Ag85A should be investigated as a new adjuvant for influenza vaccines.

## Conclusions

The pEGFP/Ag85A-HA2 vaccine induced Th1 type responses in lungs and splenocytes of inoculated mice. Increase survival after IAV infection and reduced bacterial load after *S*. *aureus* challenge was associated with vaccine-induced TLR2 expression. Altogether, our findings indicate that Ag85A could enhance immune responses to HA2 and should be investigated as a new adjuvant for influenza vaccines.

## Material and methods

### Construction of eukaryotic expression plasmid vaccine and preparation of the heat-inactivated influenza A/PR/8/34 vaccine

pEGFP/Ag85A was constructed by first cloning the Ag85A sequence (1–250 amino acids of N-terminus) [24,25] from BCG, and pEGFP/HA2 was constructed by cloning the HA2 sequence (360–530 amino acids of HA N-terminal) from the IAV strain A/PR/8/34, H1N1 as described previously [26]. These sequences contain the immunodominant epitopes of Ag85A and HA2. The hydrophilicity of the proximal 250 amino acids of Ag85A is similar to that of the HA N-terminal amino acids 290–321 [27]. Ag85A and HA2 were fused (designated as Ag85A-HA2) with a 5 glycine linker as described previously [6,28]. The pEGFP/Ag85A-HA2 was constructed by inserting Ag85A-HA2 into pEGFP-C2 (Clontech, USA). All inserted fragments were confirmed by restriction endonuclease digestion. Sequencing was performed using an ABI Prism 377XL DNA sequencer (Invitrogen, China). Large scale extraction and purification of pEGFP/Ag85A-HA2, pEGFP/Ag85A and pEGFP/HA2 were performed using the EndoFree Plasmid Maxi kit (Qiagen, Germany).

Preparation of inactivated influenza A/PR/8/34 (iPR) virus was carried out by heating at 60°C for 1 h as described previously [28]. The vaccine was stored in −20°C before use.

### Cell transfections

HEK293 cells (CRL-1573™) were obtained from the American Type Culture Collection (ATCC) Purified eukaryotic expression plasmids pEGFP/Ag85A-HA2, pEGFP/Ag85A, pEGFP-C2 and pEGFP/HA2 were transfected into HEK293 cells by using Lipofectamine™ 2000 transfection reagent (Invitrogen, China) according to the manufacturer’s instructions. Each plasmid was transfected in triplicate wells, and PBS was applied as a mock transfection control. Plasmid expression in all cell groups was assessed by fluorescence microscopy 48 h after transfection.

Total RNA of all groups was isolated as recommended by the manufacturer (Transgene, China). Contaminating genomic DNA was then eliminated from the RNA preparations by using RNase-free DNase I (Biotake, China). RT reactions were performed with EasyScript First-Strand cDNA Synthesis SuperMix (Transgene, China). cDNAs were amplified by PCR using 2× TransTaq High Fidelity (HiFi) PCR SuperMix (Transgene, China) in a 50 μl reaction mixture in accordance with the manufacturer’s recommendations. The following PCR primer pairs were used: mouse GAPDH (531), 5^′^-AAAACCTGACCTCCCTTGA-3^′^ and 5^′^-GTGCAGCCTGGTGACATT-3^′^; pEGFP/Ag85A-HA2, 5^′^-CCCTCGAGCATGCAGCTTGTTGACAG-3^′^ and 5^′^-CGCGGATCCTCATATTTCTGAAATTCTAAT-3^′^; pEGFP/Ag85A, 5^′^-CCCTCGAGCATGCAGCTTGTTGACAG-3^′^ and 5^′^-CGGGATCCTTAGCCGTTGCCGCAGTACAC-3^′^-pEGFP/HA2, 5^′^-CCGCTCGAGTATTGAAGGGGGATGGA-3^′^ and 5^′^-CGCGGATCCTCATATTTCTGAAATTCTAAT-3^′^. Control PCRs using GAPDH primers were performed on cDNA from the pEGFP/Ag85A-HA2, pEGFP/Ag85A, pEGFP-C2 and pEGFP/HA2 groups. Test PCRs of the pEGFP-C2 group were performed with pEGFP/HA2 primers, pEGFP/Ag85A primers and pEGFP/Ag85A-HA2 primers. Test PCRs of the pEGFP/Ag85A-HA2, pEGFP/Ag85A and pEGFP/HA2 groups were performed with their respective primers. All PCRs were performed simultaneously in the same conditions: 5 min at 95°C, followed by the appropriate number of cycles consisting of 30 sec at 95°C, 30 sec at 50°C and 2 min at 72°C. After amplification, 10 μl of each PCR product was electrophoresed on a 2% agarose gel and visualized by ethidium bromide staining. PCR amplification and sequencing were performed using an ABI Prism 377XL DNA sequencer (Invitrogen, China).

Cells of all group were lysed in RIPA sample buffer (Boster Biological Technology) per the manufacturer’s instructions, and supernatants were collected to analyze expression of plasmids. The samples were separated by SDS-PAGE (10%), and the proteins were transferred to PVDF membranes, which were blocked for 2 h in 5% nonfat dry milk in TBST (TBS containing 0.05% Tween-20) and hybridized at 4°C overnight in TBST with the primary antibodies (anti-HA, Catalog Number: 11055-RP02, Sino Biological Inc, china; anti-Ag85A, Catalog Number: ab14073, Abcam, UK). After washing the membranes three times in TBST, for 15 min each time, they were incubated with 1:10,000 dilutions of HRP-conjugated secondary antibodies (Boster Biotechnological). The membranes were washed three times in TBST, for 15 min each time, and the blots were visualized with an electrochemiluminescence (ECL) system (Boster Biological Technology).

### Influenza infection and post-influenza pneumonia induced by *S*. *aureus* challenge

A total of 84 female BALB/c mice, aged four to six weeks old, were purchased from the animal care center of Sichuan University and divided into 6 groups, with 14 mice in each group. They were bred under pathogen-free conditions. Each group of mice was injected separately with one of the following: pEGFP/Ag85A-HA2, pEGFP/Ag85A, pEGFP/HA2, pEGFP/c2, PBS, iPR. The mice were immunized by intramuscular injection of the quadriceps of both legs (50 μg in 50 μL PBS for each leg) twice with a 3 week interval between injections. iPR preparations were diluted 1:2 in PBS before mice were injected with 50 μL of the inoculum. Mice in the positive control group were also injected intramuscularly with 50 μL of the iPR inoculum. Body weight was monitored daily, and mice losing more than 25% of their initial weight were sacrificed and scored as dead. All experiments were performed with institutional approval.

Seven days after the booster immunization, the mice were challenged with IAV, as described previously [12,23]. In brief, influenza A/PR/8/34 was harvested from 10- to 11-day-old embryonated chickens eggs and titrated in a plaque assay. Mice were anesthetized by inhalation of isoflurane and challenged with IAV via intranasal inoculation of two 50% mouse lethal doses (MLD50) of PR8 virus diluted in PBS in a total volume of 50 μL.

After IAV challenge, the numbers of survival mice in all groups were showed in Table 2. Staphylococcal pneumonia was induced 14 days after IAV infection by intranasal inoculation of 50 μl normal saline containing 2 × 104 colony forming units (cfu) of *S*. *aureus* (ATCC 25923) as described previously [12,23]. The survival rate was monitored from day 3 after infection with S. aureus.

**Table 2:** The survival mice numbers in all groups after IAV challenge

**Group**	**iPR**	**pEGFP/Ag85-HA2**	**pEGFP/HA2**	**pEGFP/Ag85**	**PBS**	**pEGFP-C2**
number	10	10	10	9	6	7

### Specimen collection

Specimen collection was performed according to the method reported by Zhang et al. [26] and Tamura et al. [29]. In brief, the first immune serum samples were obtained from each mouse via tail bleeding 1 day before the booster immunization. The second immune serum samples were obtained from four randomly chosen mice of each group by terminal cardiac bleeding under anesthesia with chloroform at 8 days after the virus challenge. Spleens from the pEGFP/Ag85A group, pEGFP/c2 group, PBS group and iPR group were collected, and isolated splenocytes were cultured. The spleens from the pEGFP/Ag85A-HA2 and pEGFP/HA2 groups were collected, and isolated splenocytes were cultured. The supernatants of the splenocyte cultures were collected to measure cytokine production. The trachea and left lungs were collected and washed twice with 2 mL PBS containing 0.1% BSA. The bronchoalveolar lavages were centrifuged to remove cellular debris and were used for virus titration. The right lungs were homogenized at 4°C in five volumes of sterile isotonic saline with a Dounce homogenizer. The lung homogenates were collected for cytokine measurements.

For *S*. *aureus* titration, Western blotting and cytokine measurements, spleens and lungs were collected from four randomly chosen mice of each group under anesthesia with chloroform at 48 h after S. aureus challenge. The lungs were harvested and homogenized at 4°C in five volumes of sterile isotonic saline with a Dounce homogenizer. The lung homogenates were collected for S. aureus titration and cytokine measurements. The collected spleen samples were lysed in RIPA sample buffer (Boster Biotechnological, China) as described by manufacturer’s instructions, and supernatants were collected to analyze expression of TLR2.

### Antibody assay

The HA inhibition (HI) assay was performed according to the method reported by Zhang *et al*.[26]. In brief, the sera were two-fold serially diluted with PBS. Four HA units of virus (A/PR/8/34, H1N1) in 25 μl per well were added to a 96-well microtiter plate containing 25 μl of diluted serum. Chicken erythrocytes in 50 μl 0.5% (v/v) were added to each well after being mixed and incubated at 4°C for 1 h. The plate was incubated at 4°C for 30–45 min. The HI end-point titers were determined as the reciprocal of the highest serum dilution that completely inhibited hemagglutination.

The neutralisation assay was performed as previously reported [6]. In brief, the sera from day 1 before the booster immunization and day 8 after the virus challenge were collected for neutralisation assay. 50 μl 2-fold serial dilutions of treated sera were mixed with 50 μl five 50% tissue culture infective doses (TCID50) of PR8 virus and incubated for 45 min at 37°C. After washing with PBS, Madin–Darby canine kidney (MDCK) cells (ATCC, CCL-34) were inoculated with the 100 μl virus-antibody mixtures. Following a 45-min incubation, DMEM supplemented 2 μg/ml trypsin (Boster Biotechnological, China)) and 3% bovine serum albumin (Beijing Biosynthesis Biotechnology Co. LTD, China) was added to each well of a 24-well plate. Each concentration was repeated in four wells. The cytopathic effect (CPE) was assayed after incubated at 37°C for 2 days. The titer of each sample, recorded as the dilution that reduced 50% of the CPE, was calculated by the Reed–Muench method.

### Cytokine measurements

Spleens were collected 8 days after the virus challenge. Spleens were removed aseptically and processed as follows. Cells were segregated, washed, adjusted to a concentration of 5 × 106 cells per mL, and grown in a 96-well flat-bottom plate (5 × 105 cells per well) in RPMI 1640 medium, supplemented with HEPES (N-2-hydroxyethylpiperazine-N’-2-ethanesulfonic acid), glutamine and 10% heat-inactivated fetal calf serum (FCS). Cells were stimulated with 0.5 μg concanavalin A (ConA) or 1.25 μg recombinant HA of A/PR/8/34 (Catalog Number: 11684-V08H, Sino Biological Inc, china) separately. Cells were incubated at 37°C in a humidified CO2 incubator, and supernatants were harvested after 72 h. Supernatants were pooled and stored frozen at −20°C until assay. Experiments were repeated three times, and the results of one experiment are shown.

Lung homogenates were collected on day 8 after infection with A/PR/8/34 and day 2 after infection with *S*. *aureus*. For cytokine measurements, lung homogenates from each group were diluted 1:2 in RIPA buffer (Boster Biotechnological, China). Homogenates were centrifuged at 1500 × g for 15 min at 4°C as described as instructed by the manufacturer of the RIPA buffer, and supernatants were pooled and stored at −20°C until assays were performed.

IFN-γ and IL-10 from lung homogenates and IFN-γ from supernatants of splenocyte cultures were measured using specific enzyme-linked immunosorbent assays (ELISA; Boster Biotechnological, China) in accordance with the manufacturer’s recommendations.

### IAV titrations

The IAV titer assay was performed according to the method reported by Zhang et al. [26]. In brief, the bronchoalveolar lavage fluid of 8 days after the virus challenge was 10-fold serially diluted. The bronchoalveolar lavage dilutions were inoculated on confluent monolayers of Madin–Darby canine kidney (MDCK) cells (ATCC, CCL-34) in 24-well plates. Each concentration was repeated in four wells. The cytopathic effect (CPE) was assayed after incubated at 37°C for 2 days. The virus titer of each specimen, recorded as the 50% tissue culture infection dose (TCID50), was calculated by the Reed–Muench method. The virus titer in each group was presented as the mean ± SD of all specimens in each group.

### *S*.*aureus* titration

Whole lungs were collected from four randomly selected mice by cardiac bleeding under anesthesia with chloroform at 48 h after *S*. *aureus* challenge. The whole lungs were harvested and homogenized at 4°C in five volumes of sterile isotonic saline with a Dounce homogenizer. Serial 10-fold dilutions in sterile isotonic saline were made from whole lung homogenates, and 50 μL volumes were plated onto Luria-Bertani (LB) agar plates. LB agar plates were incubated at 37°C, and the colonies were counted after 16 h to determine the cfu.

### Western blotting detection of TLR2

Spleens were lysed in RIPA sample buffer (Boster Biological Technology) per the manufacturer’s instructions, and supernatants were collected to analyze expression of TLR2 at 48 h after S. aureus challenge. The samples were separated by SDS-PAGE (10%), and the proteins were transferred to PVDF membranes, which were blocked for 2 h in 5% nonfat dry milk in TBST (TBS containing 0.05% Tween-20) and hybridized at 4°C overnight in TBST with the primary antibodies (Boster Biological Technology) (anti-TLR2,1:400). After washing the membranes three times in TBST, for 15 min each time, they were incubated with 1:10,000 dilutions of HRP-conjugated secondary antibodies (Boster Biotechnological). The membranes were washed three times in TBST, for 15 min each time, and the blots were visualized with an electrochemiluminescence (ECL) system (Boster Biological Technology).

### RT-PCR detection of TLR2

Reverse transcription and PCR amplification of the TLR2 transcript were performed as described by the manufacturer (Transgene, China) of the EasyScript First-Strand cDNA Synthesis SuperMix and 2× TransTaq High Fidelity (HiFi) PCR SuperMix in a 50-μl reaction mixture. The PCR primers for mouse GAPDH (531) were 5^′^-AAAACCTGACCTCCCTTGA-3^′^ and 5^′^-GTGCAGCCTGGTGACATT-3^′^. The PCR primers for mouse TLR2 (410) were 5^′^-TTTGCTCCTGCGAACTCC-3^′^ and 5^′^-CAGCTTAAAGGGCGGGTC-3^′^. PCRs of pEGFP/Ag85A-HA2, pEGFP/Ag85A and pEGFP/HA2 groups were performed with GADPH primers and TLR2 primers. All PCRs were carried out simultaneouly in the same conditions simultaneouly. For all PCRs, the conditions were 5 min at 95°C, followed by 20 cycles consisting of 30 seconds at 95°C, 30 seconds at 60°C, and 2 min at 72°C. After amplification, 10 μl of each PCR product was electrophoresed on a 2% agarose gel and visualized by ethidium bromide staining. PCR amplification and sequencing using an ABI Prism 377XL DNA sequencer (Invitrogen, China).

### Statistical analysis

Data are presented as means ± SD. Group–group comparisons were analyzed using t-tests. Survival rates were determined using the Kaplan-Meier method. P values < 0.05 were considered significant.

### Approval of the ethics committee

All mouse experiments were performed in strict accordance with China legislation on animal experiments and approved by the Ethical Review Committee of Society for Laboratory Animal of Sichuan province (SLAS).

## Competing interests

The authors declare that they have no competing interests.

## Authors’ contributions

JD carried out most of the experiments and wrote the manuscript. DP, BW, LR, BZ, KC, and SL did part of the experiment and participated in manuscript preparation. JS, HL and YK participated in antibody detection and lung virus titration. ZJ participated in its design and coordination. ML was the main designer of the experiment and revised the manuscript. All authors read and approved the final manuscript.
